# Understanding dementia in the Western Pacific: a region-specific approach to prevention

**DOI:** 10.1016/j.lanwpc.2024.101202

**Published:** 2024-09-16

**Authors:** Jessica Gong

**Affiliations:** a Department of Epidemiology and Public Health, University College London, London, UK; b George Institute for Global Health, Imperial College London, London, UK

The Western Pacific region (WPR) is characterised by its broad spectrum of socioeconomic and ethnic groups, diverse geopolitical and natural environments, and a wide array of health and social care systems. The region's socio-historical background is particularly distinctive, shaped by significant migration patterns and rich sociocultural diversity. As the WPR's populations age rapidly, mirroring global trends, they face distinct challenges. Currently, over 245 million individuals aged 65 and older reside in WPR, a figure expected to double by 2050.^1^

Global Burden of Disease studies reveal significant variations in the projected increase in dementia cases across the WPR's countries and sub-regions^2^: Japan is expected to experience the smallest increase in dementia cases between 2019 and 2050, at 27%, given its aged population; while Mongolia is projected to see the largest rise, with an increase of 389%. In 2016, the WHO reported that 16 million individuals in the WPR were living with dementia, and by 2040, this number is expected to double in at least 10 countries within the region.^3^

In a recent comprehensive review published in *The Lancet Regional Health – Western Pacific*, Clarke and colleagues examined the prevalence of established dementia risk factors in the WPR.^4^ This analysis provides critical insights for public health strategy development and resource allocation, tailored to the specific needs of countries and the region as a whole. The review highlighted that the prevalence of several modifiable risk factors, such as alcohol consumption and diabetes, exceeds the global average in the WPR, underscoring significant areas of concern. Additionally, limited educational attainment remains pervasive, with 20% of children under 10 years old in the WPR unable to read or comprehend basic text.^4^ Other pressing issues include high obesity rates and physical inactivity: over 60% of the population in Australasia and Pacific Island countries classified as overweight or obese in 2014, and between 41% and 62% of the population in the Pacific Islands leading entirely sedentary lifestyles.^4^

The 2024 update of the Lancet Commission report on dementia risk factors further included two additional risk factors — high levels of low-density lipoprotein cholesterol and visual loss — building upon those previously identified.^5^ Some of the largest increase in non high-density lipoprotein (HDL) cholesterol from 1980 to 2018 were seen in several WPR countries, with notable sex differences.^6^ This inclusion underscores the evolving understanding of dementia risk factors, and the need to strengthen risk factor monitoring. However, many of these recognised factors are likely to disproportionately impact specific populations within the WPR, especially in underserved communities. For instance, certain risk factors contribute to greater dementia risks among First Nation populations in Australia^7^ Māori and Pacific Islanders in New Zealand,^8^ compared to majority populations in their respective countries.

Taking a broader perspective, considering the region's unique historical context — including colonisation, conflicts, displacement, and migration — warrants further investigation into how these sociohistorical determinants, and the resulting issues such as loss of cultural connectivity, trauma and adversity, in turn contribute to dementia risk through the life course in disadvantaged populations.^4^ Additionally, the implications of emerging risk factors associated with the wider environment, such as rapid urbanisation, economic growth, as well as climate threats, should be closely examined to better understand their impact on lifestyle factors, healthcare access, together with biological mechanisms (i.e., epigenetic changes)^4^ in diverse populations.

As Clarke and colleagues alluded,^4^ biological differences in terms of genetic and molecular risks on dementia vary across ethnic groups, yet research in these areas remains limited. Within the WPR, the frequency of the APOE ε4 allele varies both between and within countries among different ethnic groups. A large U.S.-based population study found that APOE ε4 confers a higher dementia risk for East Asians compared to other ethnicities, with no protective effect observed for APOE ε2.^9^ A major challenge in the WPR is the significant data gap in certain underserved communities and ethnic minority groups. Additionally, genome-wide association studies have predominantly focused on Caucasian populations of European descent, leading to significant gaps in understanding the genetic risks in diseases for other ethnic groups. This underscores the need for a better understanding of the phenotypic influence of the APOE in diverse populations, particularly regarding variations in molecular risk.^4^

With biomarker technologies continue to advance and are increasingly utilised in diagnostic tools and disease prediction models, it is crucial to evaluate the accuracy of these tests — such as p-tau-217 for dementia diagnosis — across diverse ethnic groups before they are implemented on a broader scale. With potentially disease-modifying treatments and prevention programs for dementia becoming more accessible, regulatory bodies in the WPR must ensure equitable access across diverse populations. Moreover, it is essential to recognise that the worldwide FINGERS trials, which focus on dementia prevention, have primarily been conducted in upper-middle to high-income countries, including China, Australia, Japan, South Korea, and Singapore.^10^ Expanding these prevention trials to other countries and communities is essential to meet the needs of a broader population.

Furthermore, there is a strong impetus to develop and implement national action plans for addressing dementia in WPR countries. To date, only South Korea, Japan, Australia, and Aotearoa New Zealand have such plans in place.^4^ Tackling this issue will require concerted efforts from a wide range of stakeholders, including national and local health ministries, academic institutions, international organisations, regulatory bodies, and the private sector. Additionally, neighbouring higher-income countries must engage in more robust geopolitical co-development and collaboration. Such a coordinated approach is vital for effectively addressing the challenges posed by ageing populations in the WPR and ensuring that solutions are inclusive, sustainable, and equitable across all communities.

Taken together, there is a humanitarian imperative to respond swiftly and decisively. The WPR, confronting the challenges of an ageing population alongside unique issues such as climate change and rapid economic development, offers a compelling case study of the global phenomenon of population ageing in addressing neurodegeneration. Clarke and colleagues emphasise the importance of a comprehensive, whole-of-society approach that is both adaptable and focused on meeting the specific needs of the region.^4^ The region's experience highlights the complexities and opportunities involved in managing an ageing population during significant economic and social change. Although countries in the WPR are making progress through the successful implementation of comprehensive strategies, their experiences demonstrate that achieving meaningful outcomes requires significant time, dedication, and intersectoral collaboration, as well as effective negotiation and advocacy.

## Declaration of interests

JG is supported by the 10.13039/100000049National Institute on Aging (NIA) (grant Number [R01AG17644]).
